# Vibration, temperature, and humidity influence the perception of electrocutaneous stimulation for occupational warning

**DOI:** 10.1038/s41598-025-25166-2

**Published:** 2025-11-04

**Authors:** Eva-Maria Dölker, Daniel Gröllich, Martin Schmauder, Jens Haueisen

**Affiliations:** 1 Institute of Biomedical Engineering and Informatics, Technische Universität Ilmenau, 98693 Ilmenau, Germany; 2 Institute of Material Handling and Industrial Engineering, Chair of Ergonomics, Technische Universität Dresden, 01307 Dresden, Germany

**Keywords:** Biomedical engineering, Occupational health

## Abstract

Electrocutaneous stimulation can be used to warn workers in hazardous situations. To determine parameters for the operating ranges of such warning systems we investigated the influences of vibrations, temperature, and humidity on the perception, attention, muscle twitch, and intolerance thresholds of healthy volunteers in a multi-center study. In a study on 94 participants, vibrations with amplitudes of 2 mm, 5 mm, and 8 mm and at frequencies of 8 Hz and 9.5 Hz were applied to the right arm. In a second study, 52 participants experienced temperature and humidity variations within a climate chamber in four conditions: dry cold (13.8 ± 1.6 $^{\circ }$C, 33.9 ± 9.2% relative humidity (RH)), dry warm (41.3 ± 1.7 $^{\circ }$C, 24.6 ± 5.8% RH), wet cold (12.0 ± 1.6 $^{\circ }$C, 88.5 ± 8.2% RH), and wet warm (41.3 ± 2.1 $^{\circ }$C, 65.5 ± 9.0% RH). In both studies, the upper right arm was electrically stimulated with electrodes of size 25 mm$\times$40 mm, and thresholds were determined. Perception, attention, and intolerance thresholds increased with vibration amplitude and frequency from median values of 5 mA, 9.9 mA and 19.3 mA for perception, attention and intolerance thresholds during rest to 14.6 mA, 20 mA and 21 mA during vibration amplitude of 8 mm and frequency of 9.5 Hz. Perception thresholds slightly increased with decreasing temperature with median values of 4.1 mA during dry cold vs. 3.8 mA during dry warm condition and 4.0 mA during wet cold vs. 3.8 mA during wet warm condition for an electrode pair at a lateral position. Muscle twitch thresholds slightly increased with increasing temperature at the lateral electrode pair with median values of 17.4 mA during dry cold vs. 18.8 mA during dry warm condition and 17.5 mA during wet cold vs. 18.7 mA during wet warm condition. For both studies, women showed smaller perception and attention thresholds. For illustration, in study 1, the median values of the perception threshold during rest were 4.1 mA for women and 5.7 mA for men, whereas the median values for the attention threshold were 9.1 mA for women and 11.4 mA for men, respectively. Additionally, women experienced less muscle twitching. To exemplify this, 50% of the women experienced muscle twitches compared to 66% of the men at a lateral electrode pair in study 2. We conclude that the operating range of future electrical warning systems needs to be flexibly adjusted to the working conditions. The influence of the climate conditions was minor, suggesting that electrocutaneous warning systems can operate robustly across diverse environments. This supports their practical applicability and motivates future research on optimized dry or textile-based electrodes to enhance usability in real-world settings.

## Introduction

In the realm of occupational safety, ensuring the reliable alerting of workers to potential hazards is of paramount importance. The current reliance on auditory^1^ or visual^2^ cues for warnings can prove inadequate in environments characterized by high noise levels or limited visibility. Consequently, we propose developing a warning system that utilizes electrocutaneous stimulation via textile electrodes directly integrated into the user’s attire.

Electrocutaneous stimulation is commonly employed in medical applications such as prosthetics to provide sensory feedback^3–6^. However, its adaptation for occupational safety necessitates the exploration of various stimulation parameters, highlighting the importance of foundational investigations. These baseline studies aim to reduce the parameter space, marking the initial phase of development for the electrical warning system, aligned with the aforementioned enduring objectives.

In a previous study ($n=81$ participants)^7^ we defined three thresholds relevant for electrical warning: (1) a just noticeable stimulus defines the perception threshold $A_\text {p}$, (2) a stimulus drawing attention to itself defines the attention threshold $A_\text {a}$, and (3) a stimulus generating intolerable perceptions defines the intolerance threshold $A_\text {i}$. We investigated these thresholds, qualitative and spatial perceptions in dependence of varying pulse widths, electrode sizes, and electrode positions. All threshold values decreased with increasing pulse width. For electrode sizes between 15 mm$\times$15 mm and 40 mm$\times$40 mm, perception thresholds increased with increasing electrode sizes. Notably, knocking emerged as the predominant perception for perception and attention thresholds, while muscle twitching, pinching, and stinging were predominantly reported at the intolerance threshold. Furthermore, the stimulation was localized within the region of the electrode pair employed.

Previous studies^7,8^ investigated parameters of electrocutaneous stimulation during rest and constant laboratory conditions with temperatures around $23^\circ$C. However, external environmental influences in the work environment have an impact on human perception^9^. In a preliminary investigation, we observed that the perception thresholds increased if the participant worked with a polishing machine. It is known that nerve conduction velocity decreases over time in cold environments^10^. Furthermore, correlations between cold and joint electrical pain threshold^11^ were shown. Also, heat exposure affects tactile perception thresholds^12^. Moreover, it is known that the perceived vibration sense threshold is affected by skin temperature and room temperature^13^. Thus, this study aims to investigate the thresholds for electrocutaneous stimulation under the influence of vibration, temperature, and air humidity. The parameters were chosen to mimic working conditions and the investigations were carried out in a laboratory environment to ensure reproducibility. Two studies were performed. In the first study ($n=94$ participants) the influence of vibrations on the thresholds $A_\text {p}$, $A_\text {a}$, $A_\text {i}$, and on the perception of a complex warning signal was investigated. The second study ($n=52$ participants) investigated the influences of temperature and air humidity on the thresholds using a climate chamber.

## Methods

### Experimental setup

#### Electrical stimulation

The experimental setup closely resembled that described in our prior publication^7^. Here, we provide a brief overview; for comprehensive details, please refer to^7^. We employed a custom program developed in LabVIEW 2017 (National Instruments, Austin, TX, USA) to regulate a constant current stimulator DS5 (Digitimer Ltd, Letchworth Garden City, UK) via a PC and a multiplexer D188 (Digitimer Ltd, Letchworth Garden City, UK). This configuration allowed the activation of one of the 8 output channels, delivering stimuli to designated electrode pairs. Fourteen electrodes (reusable self-adhesive TENS electrodes, axion GmbH, Leonberg, Germany, size 25 mm $\times$ 40 mm) were positioned pairwise along the centerline between the right shoulder joint and the elbow. Electrodes were spaced circumferentially at intervals equivalent to 1/8 of the arm circumference. Each pair comprised one electrode positioned 5 mm above and another 5 mm below the centerline. Consecutively numbered electrode pairs corresponded to specific arm positions: anterior (1), lateral (3), posterior (5), and medial (7). The omission of electrode pair no. 8, corresponding to the medial-anterior position, was intentional, informed by prior findings indicating frequent muscle twitches at this site. Electrode pair no. 8 was deemed nonessential for warning signal presentation^7,8^. The biphasic rectangular current stimulation signal was characterized by parameters including amplitude $A$, pulse width $t_{\mathrm{{p}}}$, pulse interval (1/pulse frequency $f_{\mathrm{{p}}}$) and number of pulses $n_{\mathrm{{p}}}$.

#### Vibration

**Fig. 1:**
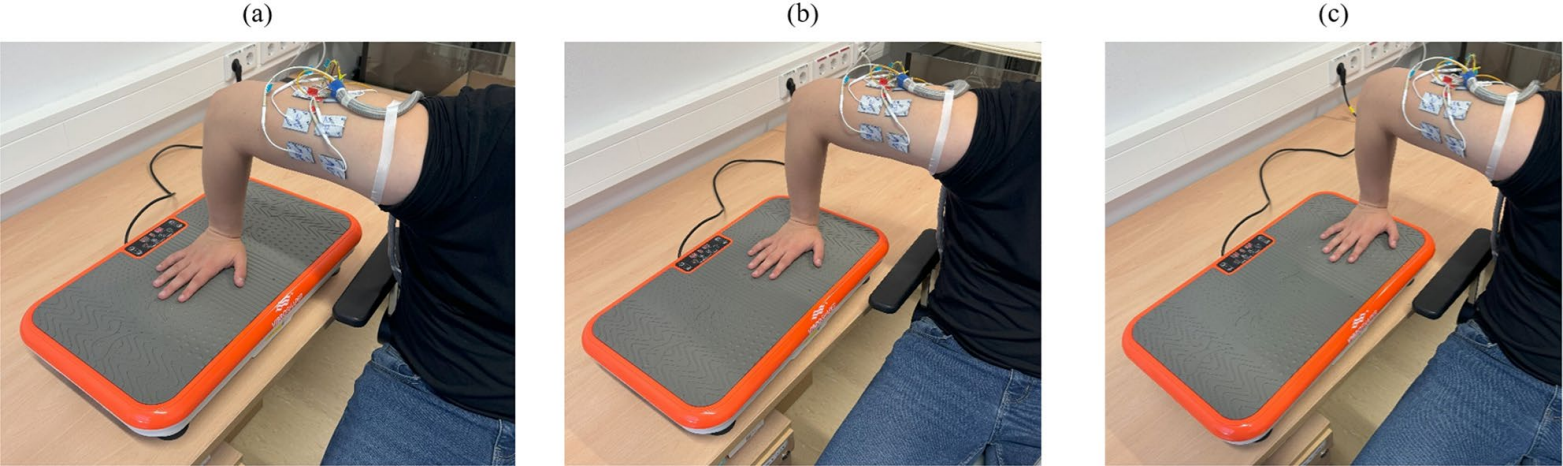
Vibration amplitudes 2 mm (**a**), 5 mm (**b**) and 8 mm (**c**) adjusted by the hand position during electrocutaneous stimulation at the upper right arm.

The vibration was transferred via the hand onto the arm using a Vibroshaper (MediaShop GmbH, Neunkirchen, Germany). The use of the Vibroshaper enables the adjustment of the vibration frequency in steps between 1 and 99 (equivalent from 8 to 13 Hz). The position of the hand influences the transferred vibration amplitude from 0 to 8 mm. The influence of the vibration on the electrocutaneous perception was investigated for the two varying frequencies 8 Hz and 9.5 Hz and the three vibration amplitudes 2 mm (cf. Fig. 1a), 5 mm (cf. Fig. 1b) and 8 mm (cf. Fig. 1c). The vibration parameters were chosen based on the capabilities of the Vibroshaper device. An amplitude of 2 mm is comparable to that of vibrating plate compactors, although such equipment typically operates at higher frequencies (30–100 Hz)^14^. Pneumatic jackhammers, by contrast, operate at lower frequencies around 23 Hz but with substantially higher displacement amplitudes of approximately 16–17 mm peak-to-peak^15^. This represents a much stronger mechanical load, which motivated the inclusion of higher vibration amplitudes (up to 8 mm) in the present study. The vibration parameters were thus not intended to replicate specific industrial machines but to model a range of mechanical intensities and frequencies relevant for studying their effects on perception thresholds under controlled and reproducible laboratory conditions. The tested frequencies (8 Hz and 9.5 Hz) were chosen to cover the device’s lower and mid-range while avoiding the upper limit of 13 Hz, which was found to be increasingly unpleasant for participants—especially at higher amplitudes.

#### Climate chamber

**Fig. 2:**
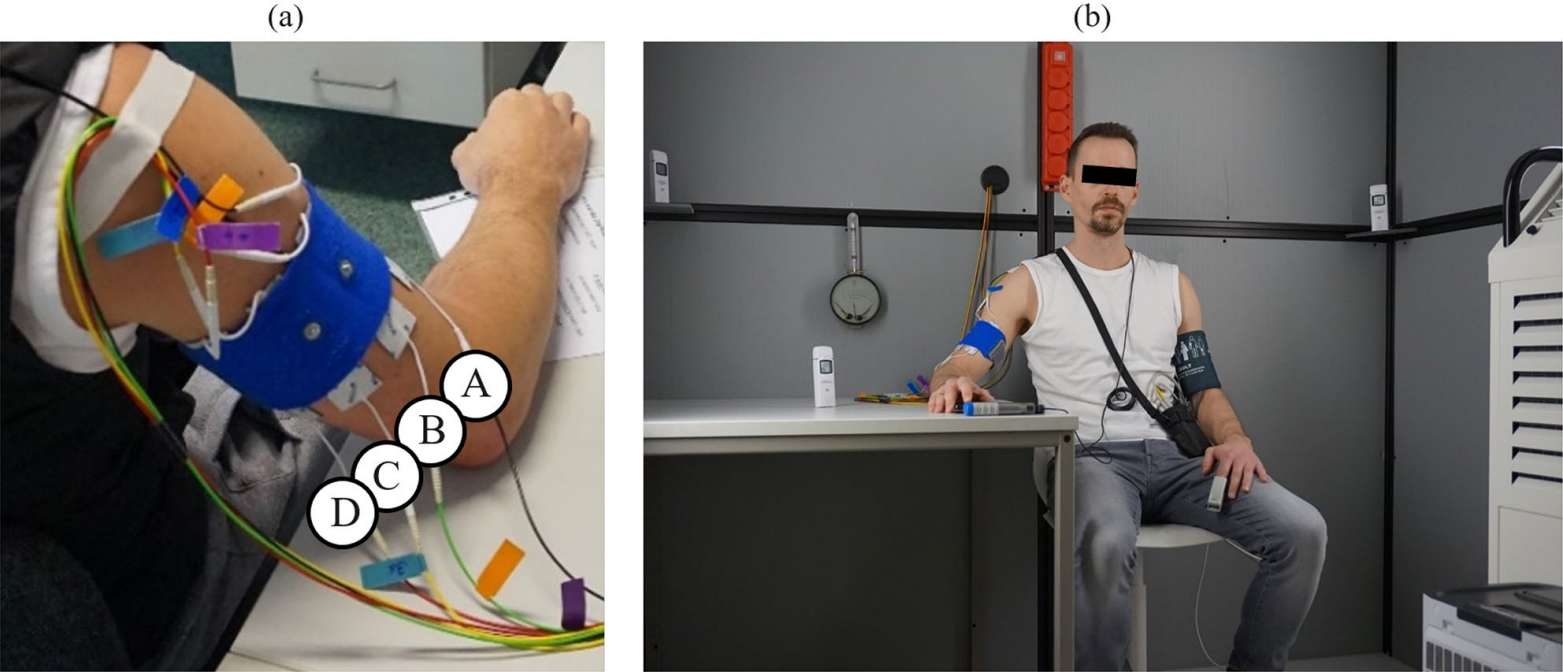
Climate chamber setup: (**a**) TENS-electrode pairs at the upper right arm fixated with a textile cuff, (**b**) Participant in the climate chamber.

In order to be able to simulate different climatic conditions, a special climate chamber (Fig. 2b) was used at TU Dresden to generate temperatures between +$10^{\circ }$C and +$45^{\circ }$C at different levels of humidity. Cold temperatures were achieved by using a PT 4500S industrial air conditioner (Trotec GmbH, Heinsberg, Germany). Fan heaters and a TIH 900 S infrared heating panel (Trotec GmbH, Heinsberg, Germany) were used to heat the climate chamber. For the simulation of very dry working environments, the ambient air in the climate chamber was dried to below 30% humidity by using a TTR 57-E dehumidifier (Trotec GmbH, Heinsberg, Germany). By using an ultrasonic humidifier Sonic 1 (AFT GmbH & Co.KG, Roßtal, Germany), a relative humidity of max. 95% was achieved in the climate chamber. All devices that were necessary to generate the stimulation signals were positioned outside the climate chamber in order to avoid device damage as well as influences by temperature and humidity. The electrocutaneous signals were transmitted via electrode cables (length 5 m) to the TENS electrodes in the climatic chamber. In order to reduce the experimental time for the participants in the climate chamber to a tolerable duration, the number of electrode pairs investigated in the climate chamber was reduced to 4 at the lateral side of the arm, where the muscle twitches are minimal^7^. The distance between the upper and lower electrodes was 2 cm. The 4 pairs of electrodes were distributed as follows: A (between electrode pair positions 1 and 2) anterior, B (between 2 and 3) and C (between 3 and 4) lateral, D (between 4 and 5) posterior on the arm (Fig. 2a). The intermediate electrode positions were chosen in order to investigate the influence of humidity and temperature on the electrocutaneous perception at the complete lateral side of the upper right arm.

### Thresholds, qualitative and spatial perceptions

In order to obtain an operational parameter spectrum for an electrocutaneous warning system, three distinct thresholds were determined as outlined in our previous study^7^: (1) the perception threshold $A_{\mathrm{{p}}}$, representing the minimum stimulus intensity perceptible to the user; (2) the attention threshold $A_{\mathrm{{a}}}$, indicating the stimulus intensity sufficient to attract attention; and (3) the intolerance threshold $A_{\mathrm{{i}}}$, signifying the stimulus intensity causing intolerable sensations. Notably, the attention threshold served as an indirect gauge of stimulation intensity required for attention-grabbing perceptions. To determine these thresholds, a single biphasic stimulation pulse with a pulse width of $t_{\mathrm{{p}}}=$150 µs was used. The amplitude was incrementally increased from 0 mA to a maximum of 25 mA in steps of 0.1 mA, 0.2 mA, or 0.5 mA, with adaptively chosen steps guided by the trained operator. To ensure consistency, the trained operator adaptively selected the step size depending on proximity to the expected thresholds, following an experiment-specific training routine. At each threshold, the operator queried the participants regarding their qualitative and spatial perceptions. Qualitative perceptions were categorized into options knocking, scratching, stinging, pain, muscle twitch, tickling, itching, pinching, and squeezing, as per questionnaire choices. It’s important to note that perceiving pain doesn’t necessarily coincide with reaching the intolerance threshold; instead, intolerance indicates that the participant deems the stimulus unbearable, while the qualitative perception chosen from the questionnaire could be stinging, pinching, or even pain. Painful sensations might occur before reaching intolerance, signifying discomfort but not intolerance. These categories align with our prior research^7,8,16^. Spatial perceptions were queried regarding positioning between electrodes, at both electrodes, specifically at the upper or lower electrode, extending beyond electrodes, or elsewhere on the body. Throughout the session, the operator indicated the threshold being assessed, enabling focused determination. Participants were briefed on threshold definitions and questionnaire categories beforehand, with any inquiries addressed prior to the experiment.

### Study 1: vibration and warning patterns

#### Study group

This part of the study was carried out at the TU Ilmenau. The descriptive statistics of the study group are listed in Table 1. The ethics committee of the Faculty of Medicine of the Friedrich-Schiller-University Jena, Germany, approved the study. All methods were carried out in accordance with relevant guidelines and regulations. All participants gave written informed consent. The sample size was not based on a formal statistical power calculation but was defined based on prior experience and feasibility considerations. In this study, more participants than initially planned were included to enhance data robustness and allow for exploratory subgroup analyses.

**Table 1:** Descriptive statistics of the study group of the vibration and warning patterns study. Age and arm circumference are given as mean ± standard deviation.

Participants	$n=94$
Sex	Female: 43 Male: 51
Age in years	24.2 ± 3.2 Youngest: 18 years Oldest: 31 years
Handedness	Right: 88 Left: 6 Both: 0
Arm circumference (right arm) in cm	30.4 ± 4.0

For the day of the experiment and the day before the experiment, participants were asked to get a sufficient amount of sleep, to not consume caffeine, nicotine, or alcohol, to drink enough (approx. 2 l), to do no hard physical work or sports, and not treat upper arms with skin cream.

#### Experimental paradigm

The entire experiment, encompassing preparation time and all sub-experiments, lasted 76±13 min. (mean±standard deviation), conducted on the same day. The duration primarily varied based on participant factors. Mean durations ± standard deviations for each sub-experiment within the paradigm are specified alongside corresponding paragraphs. These durations include preparation time for subsequent sub-experiments, typically spanning 2–4 minutes. Additionally, the final time point was recorded at the outset of the ’Warning pattern’ sub-experiment’s conclusion. Consequently, approximately 5 min. should be added to the total experiment duration, with no mean value±standard deviation available for this final sub-experiment.

*Preparation (30±0 min).* The devices were turned on 30 minutes before the experiment to allow for warm-up, with special attention given to stabilizing the current stimulator DS5. This involved connecting a load resistor (1 k$\mathrm {\Omega }$) to the DS5 and administering a series of stimulation pulses through the load resistor multiple times. To enhance the skin-to-electrode transition impedance, the participant’s right upper arm was cleaned and moistened with a wet towel. The participant was instructed to sit comfortably, relax the arm, avert gaze from the experimental setup, and focus on perceiving the stimulation. During the experiment, the arm remained in a relaxed, bent position on a table, conducted in an environment with a temperature of approximately $23^\circ$C.

*Reference threshold experiment (31±10 min).* In the reference threshold experiment at electrode pair 3, the threshold determination process was iterated ten times. This repetitive approach allowed participants to familiarize themselves with detecting thresholds and reporting perceptions, ensuring adaptation to the sensation of current stimulation. Mean values for perception, attention, and intolerance thresholds were derived from the last 3 measurement series. The most frequent qualitative and spatial perceptions across the three measurement series were selected, with the last one chosen in cases of varied perceptions. A warning amplitude *A* during rest is defined as

1 $$\begin{aligned} A = A_\text {a}+ 0.2(A_\text {i}-A_\text {a}) \end{aligned}$$

that is calculated from the determined mean values of attention threshold $A_\text {a}$ and intolerance threshold $A_\text {i}$.

*Perception thresholds under vibration (18±6 min).* The perception, attention, and intolerance thresholds were determined for electrode pair 3 under vibration with the Vibroshaper for the two frequencies 8 and 9.5 Hz and the three vibration amplitudes: 2 mm, 5 mm and 8 mm (cf. Fig. 1). A second warning amplitude $A_v$ was calculated according to eq. 1 using the attention and intolerance thresholds at vibration amplitude 8 mm and vibration frequency 9.5 Hz.

*Warning pattern.* Next, we aimed to investigate the perception of the warning signal during mechanical vibration elicited by the Vibroshaper. In a previous pilot study (n=16)^8^, an electrical stimulation signal was designed for electrical warning, referred to as the warning pattern. The pilot study revealed that an electrocutaneous stimulation signal that generates a vibrating perception and was applied as a circumferential signal around the arm showed the highest values of alertness and was therefore used as the warning pattern in this study. The previous study also indicated the necessity to use individual pulse intervals for an optimal vibrating electrocutaneous warning sensation. To determine these individual pulse intervals, five consecutive bi-phasic stimulation pulses were presented with a pulse interval of 19 ms^8^ at rest. The pulse interval was adjusted to find the optimal vibrating electrocutaneous warning sensation. This stimulation signal was then presented as the warning signal circumferentially from electrode pair 1 to 7. The signal duration was 0.4 s per electrode pair with breaks of 0.1 s between the pairs. To evaluate the warning pattern, the participants were asked to rate the perceived stimulation signal according to scales of alertness, discomfort, and urgency from 0 (no perception) over 5 (middle) to 9 (very strong). At first, the warning pattern was presented during rest without the Vibroshaper at warning amplitude *A*. The stimulation perception at rest was assigned a value of 5 for alertness, discomfort, and urgency. The participant was asked to rate all following stimulation signals relative to the warning pattern during rest. After that the warning pattern was presented with the Vibroshaper switched on (vibration frequency 9.5 Hz and vibration amplitude 8 mm, cf. Fig. 1c). The stimulation amplitude was increased from *A* (cf. eq. 1) to $A_v$ with an increment *i* of

2 $$\begin{aligned} i= {\left\{ \begin{array}{ll} 2\,\,\text {mA} & A_v-A<6 \,\text {mA}\\ 3\,\,\text {mA} & A_v-A=6-12 \,\text {mA}\\ 4\,\,\text {mA} & A_v-A>12 \,\text {mA}.\\ \end{array}\right. } \end{aligned}$$

If the warning amplitude $A_v$ determined during mechanical vibration was reached and the participant felt still comfortable enough to proceed, the amplitude was further increased by increments of 1 mA. The amplitude was increased until the participant reported an intolerable perception but maximal up to 25 mA. The signal at each amplitude was evaluated regarding alertness, discomfort, and urgency. If muscle twitching occurred it was rated regarding the strength and location. The operator & participants were given the option to choose between the following muscle twitch strength levels: none, perceivable, visible, visible & strong, arm movements, intolerable. A perceivable muscle twitch indicates the case when the participant can feel a slight muscle twitch that is not visible. As soon as this slight twitch becomes visible to the operator and the participant, the strength is indicated as visible. A visible & strong muscle twitch describes the muscle twitches of the upper arm that lead to movements just of the upper arm. The category arm movements describes movements of the whole right arm due to the muscle twitches.

### Study 2: influence of temperature and humidity

#### Study group

This part of the study was carried out at the TU Dresden. The ethics committee of the Technische Universität Dresden, Germany, approved the study. All methods were carried out in accordance with relevant guidelines and regulations. All participants gave written informed consent. The intended sample size for this study was based on feasibility considerations and aimed to be comparable to study no. 1. However, the number of participants remained below the initial target of 80 participants due to the considerable logistical and physical demands of the experimental protocol, particularly related to the climate chamber experimental paradigm. Each participant was assigned to one out of four groups with a different climate chamber schedule. The assignment is explained in the experimental paradigm section below. The descriptive statistics of the whole study group and the four groups are listed in Table 2.

**Table 2:** Descriptive statistics of temperature and humidity study for the whole study group and the subgroups 1 to 4 each participant was assigned to. Age and arm circumference are given as mean ± standard deviation. Each group corresponds to a different climate chamber schedule.

	All participants	Group 1	Group 2	Group 3	Group 4
Participants	$n=52$	$n=14$	$n=13$	$n=13$	$n=12$
Sex	Female: 20 Male: 32	Female: 5 Male: 9	Female: 3 Male: 10	Female: 7 Male: 6	Female: 5 Male: 7
Age in years	27.5 ± 5.7 Youngest: 19 Oldest: 41	26.6±5.7 Youngest: 21 Oldest: 38	26.5±5.4 Youngest: 20 Oldest: 37	27.5±6.7 Youngest: 19 Oldest: 41	29.8±4.6 Youngest: 24 Oldest: 38
Handedness	Right: 48 Left: 3 Both: 1	Right: 14 Left: 0 Both: 0	Right: 11 Left: 1 Both: 1	Right: 12 Left: 1 Both: 0	Right: 11 Left: 1 Both: 0
Arm circumference (right arm) in cm	29.4 ± 3.7	30.0 ± 4.4	28.8 ± 2.9	29.7 ± 3.7	28.8 ± 4.2

To assess comparability across the four experimental groups, statistical analyses were conducted for age, arm circumference, sex distribution, and handedness. Median age and arm circumference were compared using the Wilcoxon rank-sum test for independent samples; sex and handedness distributions were analyzed using Fisher’s exact test. All tests were two-sided with an $\alpha$ level of 0.05, and *p*-values were adjusted using the Bonferroni–Holm correction. No significant differences were found between the four groups in terms of median age, median arm circumference, sex distribution, or handedness.

The same statistical procedures were applied to compare participants from study 1 and study 2. No significant differences were observed regarding sex, handedness, or median arm circumference. However, a significant difference in age was found: the median age in study 2 was higher than in study 1. In study 1, the maximum participant age was 31 years, whereas 11 participants in study 2 were older than 31 years.

#### Climate chamber conditions

Two factors were investigated between participant groups: the order of the temperature (cold first vs. warm first) and the order of humidity (dry first vs. wet first) which led to four participant groups. Within each group, the factors temperature (cold vs. warm) and humidity (dry vs. wet) were investigated. The climatic conditions are shown in Table 3.

**Table 3:** Climatic conditions within the climate chamber during the experiment.

Climate	Temperature	Relative humidity (RH)
Constant dry-cold climate (DC)	13.8 ± 1.6 $^{\circ }$C	33.9 ± 9.2% RH
Constant wet-cold climate (WC)	12.0 ± 1.6 $^{\circ }$C	88.5 ± 8.2% RH
Constant dry-warm climate (DW)	41.3 ± 1.7 $^{\circ }$C	24.6 ± 5.8% RH
Constant wet-warm climate (WW)	41.3 ± 2.1 $^{\circ }$C	65.5 ± 9.0% RH

The temperature ranges were chosen in such a way that cold receptors are activated in the skin in cold environments and heat receptors in warm environments. This means that in addition to the electrocutaneous stimuli, also thermo-relevant information arrives at the brain^17^, which has to be processed at the same time. Tests at temperatures below +$10^{\circ }$C were not performed, as thermal protective clothing is usually worn at temperatures below this. The microclimate on the skin under the clothing is then ideally in the comfort zone. All participants were provided with standardized upper body clothing (sleeveless t-shirt and in cold environments a thermal vest).

#### Experimental paradigm

*Preparation.* Before the start of the experiment, the skin was cleaned, and if necessary hair was shaved in the area of the stimulation.

The thresholds, qualitative and spatial perceptions were determined in three different situations: habituation threshold experiment, reference threshold experiment, and within the climate chamber.

*Habituation threshold experiment* was the first threshold determination experiment outside the climate chamber, under normal environmental conditions (approximately $22^{\circ }$C and 50% RH). It consisted of at least two measurement cycles. One measurement cycle involved determining the thresholds, qualitative and spatial perceptions at the 4 electrode positions. The participant should get used to the electrocutanous stimulation and the reporting of the thresholds as well as qualitative and spatial perception.

*Reference threshold experiment.* The thresholds, qualitative and spatial perception were determined for one measurement cycle for all 4 electrode positions outside the climate chamber, under normal environmental conditions (approximately $22^{\circ }$C and 50% RH). The reference threshold experiment was repeated during the course of the experiment, cf. Table 4.

*Climate chamber.* After a ten-minute acclimatization period in the climate chamber, 3 measurement cycles were conducted to determine the thresholds as well as qualitative and spatial perceptions at all 4 electrode positions.

Threshold values, qualitative and spatial perceptions were determined for two different temperature-humidity conditions per study day. Each participant started one day in a cold environment and one day in a warm environment in order to exclude any influence of the start condition on the measured values. Humidity was kept constant per study day while temperature was adjusted (e.g. study day 1: dry-cold vs. dry-warm; study day 2: wet-warm vs. wet-cold). Table 4 shows the experimental schedule on day 1 and day 2 for the 4 groups of participants. Each participant was assigned to one of the 4 groups.

**Table 4:** Experimental schedule for four different groups of participants. Thresholds, qualitative and spatial perceptions were determined in the habituation threshold experiment (HTE), reference threshold experiment (RTE), and in the climate chamber under dry-cold (DC), dry-warm (DW), wet-cold (WC), and wet-warm (WW) conditions. Climate chamber durations include acclimatization.

		Lab 16 ± 8 min	Lab 9 ± 4 min	Climate chamber 47 ± 4 min	Lab 45 ± 12 min	Lab 9 ± 6 min	Climate chamber 47 ± 4 min	Lab 8 ± 2 min
Group 1:	Day 1:	HTE	RTE1	**DC**	Break	RTE2	**DW**	RTE3
	Day 2:	HTE	RTE1	**WW**	Break	RTE2	**WC**	RTE3
Group 2:	Day 1:	HTE	RTE1	**WC**	Break	RTE2	**WW**	RTE3
	Day 2:	HTE	RTE1	**DW**	Break	RTE2	**DC**	RTE3
Group 3:	Day 1:	HTE	RTE1	**DW**	Break	RTE2	**DC**	RTE3
	Day 2:	HTE	RTE1	**WC**	Break	RTE2	**WW**	RTE3
Group 4:	Day 1:	HTE	RTE1	**WW**	Break	RTE2	**WC**	RTE3
	Day 2:	HTE	RTE1	**DC**	Break	RTE2	**DW**	RTE3

In all measurement cycles, the amplitude value at which muscle twitching occurred, if applicable, was also documented. In addition, skin surface moisture and skin surface temperature were continuously recorded automatically at the stimulation area (right upper arm) using a P5185 USB data logger (PeakTech Prüf- und Messtechnik GmbH, Ahrensburg, Germany). The sensor was placed directly on the skin between electrode pair B and pair C.

### Statistics and analysis

The experimental results were analyzed using MATLAB 2023$\hbox {b}^{\textregistered}$ (The MathWorks, Inc., Natick, Massachusetts, USA). The results were visualized using boxplots, violin plots, and frequency distributions and quantified by median M and the interquartile range *IQR* due to skewed distributions with outliers. The thresholds in dependence of vibration frequencies and vibration amplitude as well as in dependence of temperature and humidity were compared by pairwise Wilcoxon tests. *p*-values were corrected separately by the Bonferroni-Holm procedure for each investigated threshold. *p*-values < 0.05 were considered statistically significant. Wilcoxon tests were chosen instead of repeated measures ANOVA because of skewed distributions with outliers. The thresholds were compared regarding sex using unpaired Wilcoxon tests with *p*-values corrected by the Bonferroni-Holm procedure. To investigate the ordinal scales of alertness, discomfort, and urgency of the presented electrical warning pattern, paired and unpaired Wilcoxon tests were used.

## Results

### Study 1: vibration and warning patterns

#### Perception thresholds under vibration

**Fig. 3:**
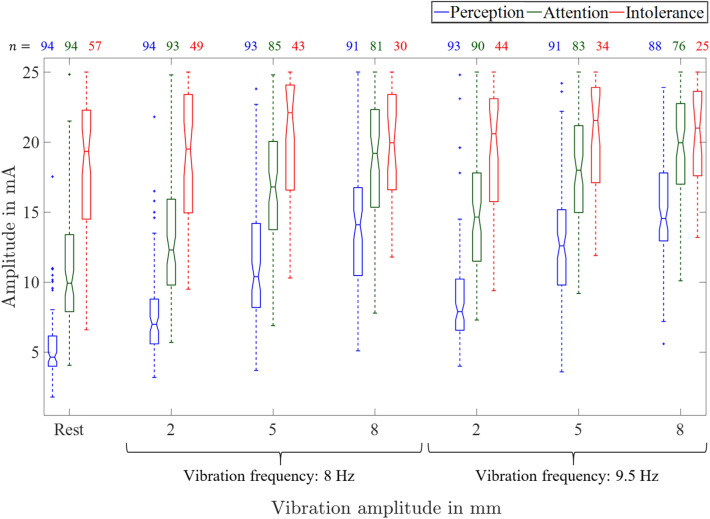
Thresholds at electrode pair no. 3 during rest and under mechanical vibration with the Vibroshaper for vibration amplitudes 2 mm, 5 mm and 8 mm and the two vibration frequencies 8 Hz and 9.5 Hz. The numbers *n* of participants reaching ($\le$ 25 mA) the perception, attention, and intolerance thresholds are given above the boxplot.

Figure 3 shows the perception, attention, and intolerance thresholds at the lateral electrode pair no. 3 during rest and under mechanical vibration conditions (cf. Fig. 1).

In Fig. 3, the solid horizontal line in the middle of each box indicates the median. If the median is not centered within the box, it shows a skewed distribution of the threshold values. The bottom and top of each box are the 25th and 75th percentiles of the sample. The distance between the bottom and the top of each box describes the interquartile range. The whiskers (dashed lines extending above and below each box) go from the end of the interquartile range to the adjacent value, which refers to the most extreme data value that is not an outlier. An outlier (cf. Fig. 3, values marked by +) refers to a value that is more than 1.5 times the interquartile range away from the bottom or top of the box. The notches display the variability of the median between samples. The width of the notch is computed such that boxes whose notches do not overlap have different medians at the 5% significance level^18–20^.

Figure 3 shows an increase of the perception (blue) to attention (green) thresholds with increasing vibration amplitude and increasing vibration frequency. Perception and attention thresholds were larger under vibration compared to rest. This effect is not visible in Fig. 3 for the intolerance thresholds from the boxplots as the notches overlap. But it can observed that the number of participants that reach ($\le$ 25 mA) perception, attention, and intolerance threshold decrease with increasing vibration amplitude and frequency. Pair-wise comparison by Wilcoxon tests showed that thresholds were larger during vibration compared to rest and that the thresholds increase with increasing vibration amplitude and frequency. All *p*-values, corrected by Bonferroni-Holm-procedure, were smaller than 0.05 except for the vibration frequency comparison of the intolerance threshold at a vibration amplitude of 2 cm ($p=0.15$).

The perception threshold was smaller in women than in men for all vibration conditions and during rest ($p<0.05$). The attention thresholds were smaller for women during rest ($p=0.04$) and at a vibration frequency of 8 Hz and vibration amplitude of 2 mm ($p=0.04$). No significant differences were found for the other vibration configurations and for the intolerance threshold. Women did reach ($\le$ 25 mA) these thresholds more often than men (cf. Table S1).

#### Warning pattern

**Fig. 4:**
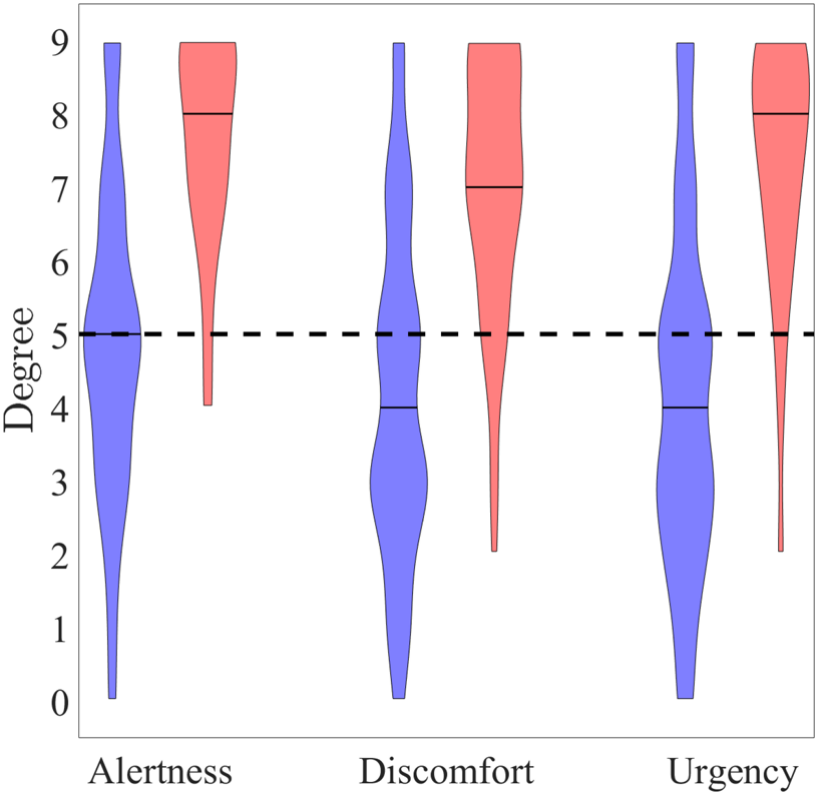
Alertness, discomfort, and urgency during warning pattern presentation at warning amplitude *A* (blue) and vibration warning amplitude $A_v$ (red). The dashed line indicates the value of 5 assigned to alertness, discomfort, and urgency for a warning pattern presentation during rest.

Figure 4 shows the rated values of alertness, discomfort, and urgency during warning pattern presentation at warning amplitude *A* (Fig. 4, blue) and vibration warning amplitude $A_v$ (Fig. 4, red). All values were evaluated relative to the warning pattern presented during rest, where values of alertness, discomfort, and urgency were assigned to the value 5 (Fig. 4, dashed line). A left-tailed Wilcoxon test for female (f) and male (m) participants with the null hypothesis that the median of alertness, discomfort, and urgency at warning amplitude *A* is larger than 5 was rejected with $p \ll 0.05$ for discomfort (f: $p=0.004$, m: $p=0.027$) and urgency (f: $p=0.047$, m: $p=0.003$) and not for alertness (f: $p=0.285$, m: $p=0.379$). The median values at warning amplitude *A* of all three scales appeared smaller compared to vibration warning amplitude $A_v$ ($p \ll 0.05$) for both sexes. Unpaired Wilcoxon tests revealed no significant differences ($p>0.05$) in alertness, discomfort, and urgency between the sexes.

**Fig. 5:**
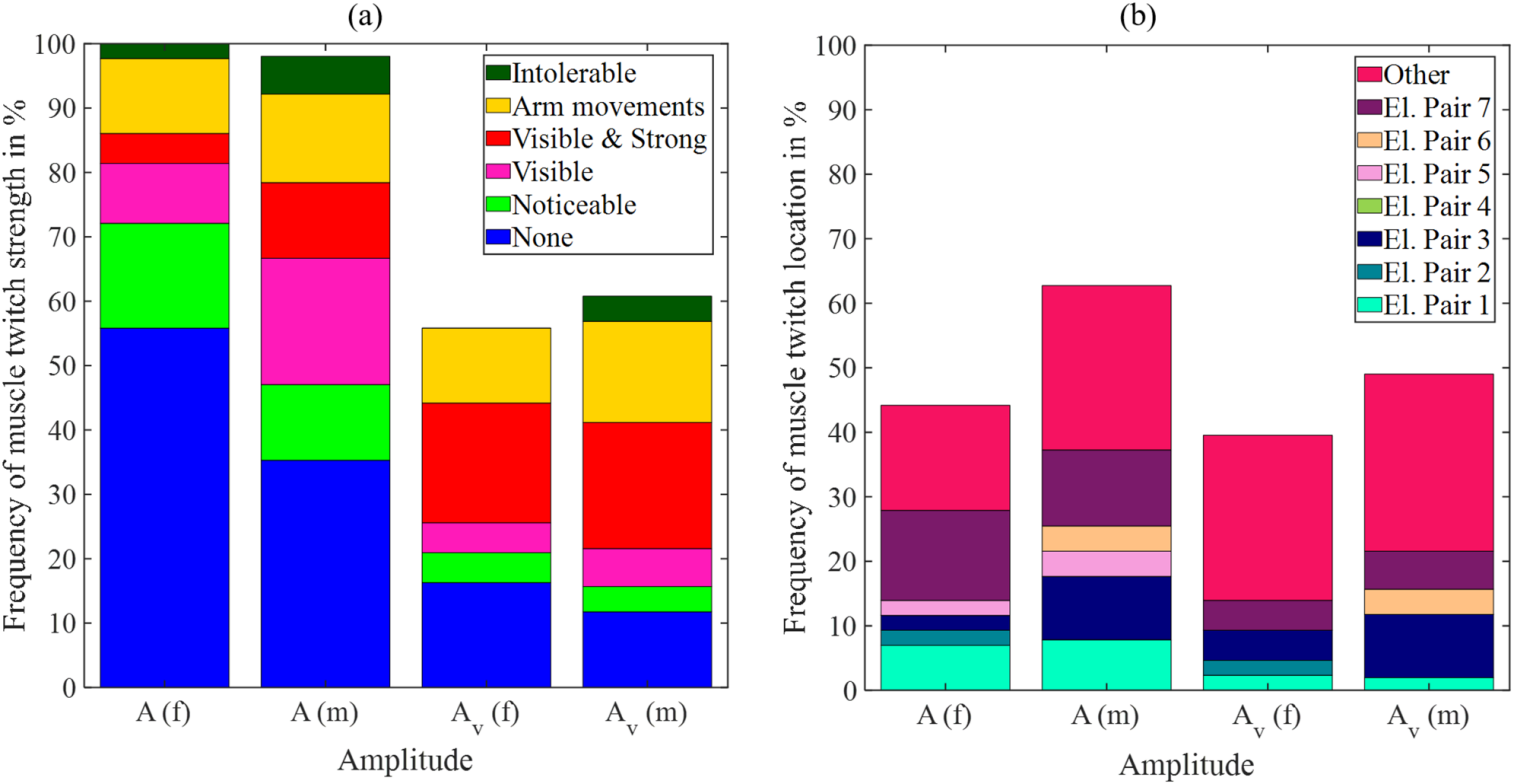
Strength (**a**) and location (**b**) of muscle twitches during warning pattern presentation at warning amplitude *A* and vibration warning amplitude $A_v$ for female (f) and male (m) participants in % according to the total amount of female and male participants.

Figure 5a shows the distribution of muscle twitch strength at warning amplitude *A* and vibration warning amplitude $A_v$ for female (f) and male (m) participants in % of the total of female and male participants. 44 % of the female participants showed muscle twitches at the warning amplitude *A*. In contrast, 65 % of the male participants showed muscle twitches at *A*. One male participant terminated this experiment before the warning pattern presentation. 40 % of the female and 37 % of the male participants terminated this experiment before reaching $A_v$ as it was too uncomfortable for them. The muscle twitch strength increased from amplitude *A* to amplitude $A_v$. Figure 5b shows the location of muscle twitches, where ’Other’ refers to the case that the muscle twitches were experienced at more than one electrode pair position. The number of muscle twitch locations at vibration warning amplitude $A_v$ (Fig. 5b, $A_v$) is reduced compared to the number of muscle twitch occurrences (Fig. 5a, $A_v$) as the corresponding participants were not able to categorize the muscle twitch location.

### Study 2: influence of temperature and humidity

#### Reference thresholds

**Fig. 6:**
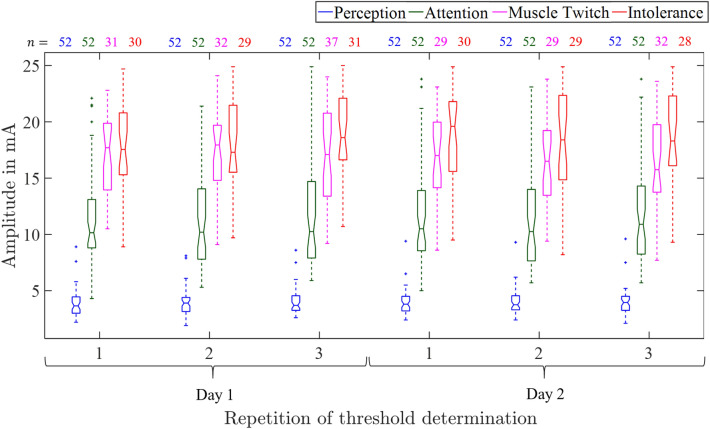
Reference thresholds at electrode pair B outside the climate chamber. The numbers *n* of participants reaching ($\le$ 25 mA) the thresholds are given above the boxplot.

Figure 6 shows the reference thresholds at electrode pair B for perception, attention, muscle twitching, and intolerance of all participants during the 2-day experiment. Thresholds were compared pair-wise using paired Wilcoxon tests within one day (e.g. $A_\text {p,Day1-1}$ vs. $A_\text {p,Day1-2}$) and between the days (e.g. $A_\text {p,Day1-1}$ vs. $A_\text {p,Day2-1}$) for each electrode pair separately. *p*-values were corrected using the Bonferroni-Holm procedure for each threshold and electrode pair. Two significant differences were found for electrode pair C at the muscle twitch threshold on day 1 comparing repetitions 1 and 2 ($p=0.01$) and for electrode pair D at the intolerance threshold during day 2 comparing repetitions 1 and 2 ($p=0.046$). Regarding sex, the unpaired Wilcoxon test showed that the perception thresholds were smaller for female than for male participants for electrode pair A. The attention threshold was smaller for women compared to men at electrode pair B: day 1, repetition 2 ($p=0.048$); day 2, repetition 2 ($p=0.048$) and repetition 3 ($p=0.03$) and electrode pair D: day 1, repetition 2 ($p=0.04$); day 2, repetition 2 ($p=0.03$). No differences were found regarding muscle twitch and intolerance thresholds. It was observed that the percentage of female participants that showed muscle twitches was smaller compared to male participants (cf. Table S2). Male participants showed the highest percentage of muscle twitches at electrode pairs A and C (cf. Table S2). This effect was not present for female participants. The percentage of participants that reached ($\le 25$ mA) the intolerance threshold was larger for female participants (cf. Table S2). Percentages of participants that reached the intolerance thresholds were larger at day 1 compared to day 2 for electrode pairs C and D (cf. Table S2).

#### Thresholds in dependence of temperature and humidity

**Fig. 7:**
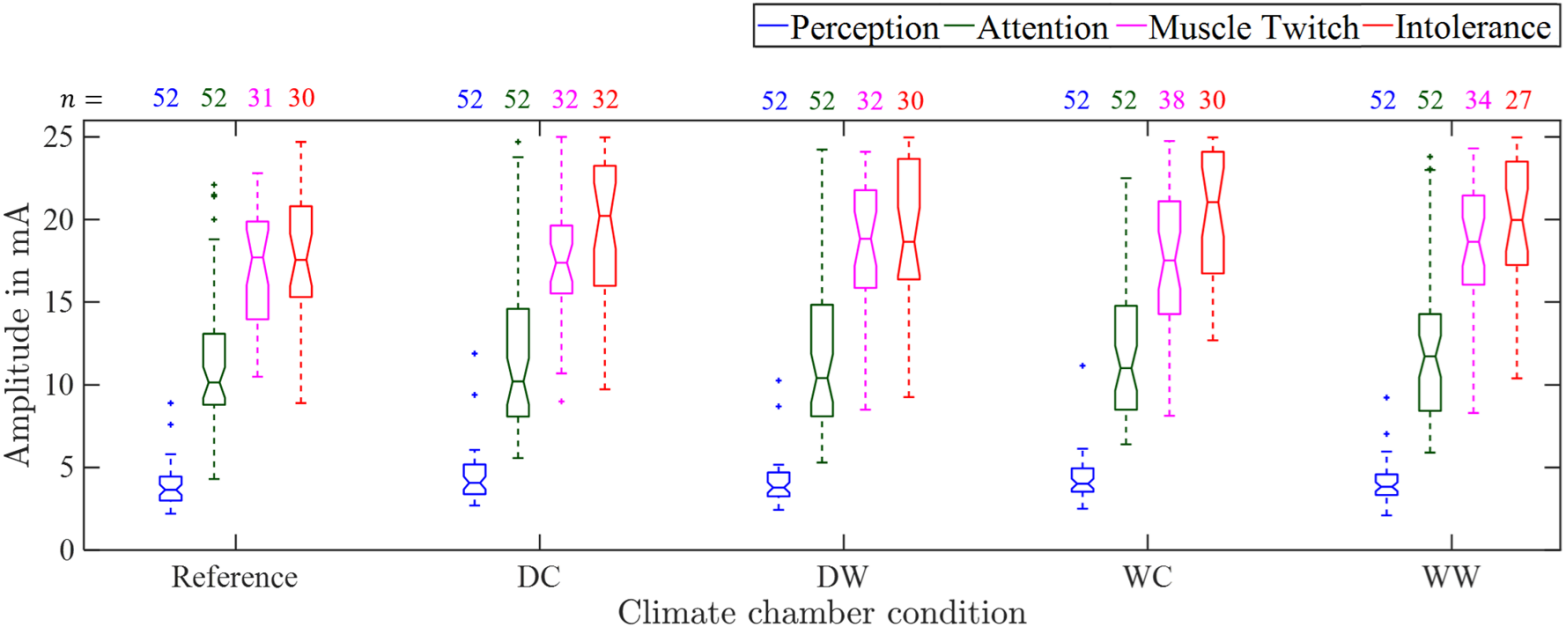
Thresholds at electrode pair B for climatic conditions outside of the climate chamber (Reference), dry-cold (DC), dry-warm (DW), wet-cold (WC), and wet-warm (WW).

Figure 7 shows the thresholds for the first reference threshold determination of day 1 and the four climatic conditions DC, DW, WC, and WW for all participants. Thresholds were compared pair-wise using paired Wilcoxon tests within one humidity (e.g. $A_\text {p,DC}$ vs. $A_\text {p,DW}$) and within one temperature (e.g. $A_\text {p,DC}$ vs. $A_\text {p,WC}$) for each electrode pair separately. *p*-values were corrected using the Bonferroni-Holm procedure for each threshold and electrode pair. The median perception thresholds were larger at lower temperatures compared to higher temperatures ($p<0.05$) except for electrode pair C with $A_\text {p,WC}$ vs. $A_\text {p,WW}$ ($p=0.21$). Fig. S1 shows the boxplots of the perception thresholds enlarged. The median muscle twitch threshold was larger at higher temperatures compared to lower temperatures. This difference was significant for electrode pair B and for electrode pair D during the dry climate condition. No changes were observed for the attention and intolerance thresholds. No significant differences were found for the thresholds comparing varying humidity.

The reference perception threshold (day 1, repetition 1) was smaller than the perception thresholds of all four climatic conditions, except for electrode pair D in condition WW ($p=0.05$). For example at electrode pair B, the median value of reference $A_\text {p}$ (day 1, repetition 1) was at 3.65 mA compared to 4.07 mA, 3.78 mA, 4.02 mA, and 3.83 mA during DC, DW, WC, and WW. The reference attention threshold was smaller than the attention thresholds of all four climatic conditions, except for electrode pair B. For example, at electrode pair A, the median value of reference $A_\text {a}$ (day 1, repetition 1) was at 9.35 mA compared to 10.01 mA, 10.85 mA, 11.5 mA, and 10.78 mA during DC, DW, WC, and WW. There was one significant difference ($p=0.004$) regarding the reference muscle twitch threshold of 17.7 mA vs. muscle twitch threshold at the DC condition at electrode pair B of 17.38 mA. The reference intolerance threshold was smaller than the intolerance thresholds of all four climatic conditions, except for electrode pair D in condition DC ($p=0.07$). For example at electrode pair B, the median value of reference $A_\text {p}$ (day 1, repetition 1) was at 17.55 mA compared to 20.22 mA, 18.65 mA, 21.05 mA, and 19.97 mA during DC, DW, WC, and WW.

The perception threshold was smaller in women compared to men for all climate conditions and during rest ($p<0.05$), except for electrode pair C at climate conditions DC ($p=0.13$), DW ($p=0.13$), and WC ($p=0.09$). The attention threshold was smaller in female than in male participants for electrode pairs A, B, and D, except condition DW for electrode pair B ($p=0.05$) and condition DC for electrode pair B ($p=0.07$). No differences were found regarding sex for muscle twitch and intolerance thresholds.

We observed that the percentage of female participants that showed muscle twitches was smaller than the percentage of male participants (cf. Table S3). Male participants showed the highest percentage of muscle twitches at electrode pairs A and C (cf. Table S2). This effect was not present for female participants. Female participants experienced muscle twitches less often during dry climate conditions compared to wet climate conditions. For male participants, there was the trend that the percentage of muscle twitch decreased with temperature (cf. Table S2).

The percentage of participants that reached ($\le 25$ mA) the intolerance threshold was larger for female participants (cf. Table S3). The percentages of participants that did not reach the intolerance threshold was higher within the climate chamber compared to the first reference threshold determination of day 1 (cf. Table S3). There was a trend for both sexes that the percentage of participants that reached ($\le 25$ mA) the intolerance threshold increased with temperature. However, this observation was not consistent for all electrode pairs.

Skin surface temperature and moisture increased, especially in warm, humid environments due to sweating and high humidity (see Table S4).

## Discussion

Perception, attention, and intolerance thresholds increased with both increasing vibration amplitude and frequency. The vibration emitted by the Vibroshaper predominantly triggered Pacini corpuscles^21^, although other mechanoreceptors may also be activated. This activation is higher for higher vibration amplitudes and frequencies. Consequently, perception, attention, and intolerance thresholds were elevated to engage mechanoreceptors beyond mechanical vibration perception. With strong mechanical vibration interference, the participants were mostly not able to report the qualitative and spatial perception. The increase of the intolerance threshold was not directly visible in Fig. 3 but was significant with the paired Wilcoxon test. It should be noted that the number of participants that reached the intolerance threshold ($\le$25 mA) decreased with increasing vibration amplitude and frequency (cf. Fig. 3). So if two distributions, e.g. from $A_i$ at vibration amplitude of 2 mm and frequency of 8 Hz ($n=49$) and $A_i$ at vibration amplitude of 8 mm and frequency of 8 Hz ($n=30$) were compared, the Wilcoxon test considered only $n=30$ data pairs, the remaining $n=19$ values had intolerance thresholds >25 mA and were therefore not further specified. In consequence, the increase in the intolerance threshold was not immediately visible in the boxplots.

The individual operating range^7^ of the electrical warning system was expected to be between the attention and the intolerance thresholds determined for single pulses. However, only 61 % of the participants reached ($\le$25 mA) the intolerance threshold. This portion even decreased with increasing vibration amplitude and frequency to 27% (vibration amplitude of 8 mm and frequency of 9.5 Hz). However, this result does not mean necessarily that the operating range needs to be increased to amplitudes >25 mA. The individual attention and intolerance threshold determination was based on single pulses while the warning pattern consisted of multiple consecutive pulses depending on the individual pulse interval that led to a vibrating perception, e.g. for a pulse interval of 19 ms and a signal duration of 0.4 s per electrode pair, 21 bi-phasic rectangular pulses were presented at each electrode pair. In consequence, the provided signal energy was larger for the warning pattern. For 10% of the participants, the warning pattern presentation was stopped directly at the warning amplitude *A* because the stimulation was too uncomfortable. For 28% of the participants, the stimulation amplitude of the warning signal was further increased but needed to be stopped before vibration warning amplitude $A_v$ due to too uncomfortable perception. For 19% of the participants, a stimulation with an amplitude $\ge A_v$ was possible. For 43% of the participants, it was possible to present the warning signal up to 25 mA with high median [25-75th percentile] values of alertness, discomfort, and urgency of 9 [7–9], 8 [6.5–9.5], and 8 [7–9]. Given the variability of these medians and the percentages of participants reporting uncomfortable perceptions, we conclude that the use of the individual warning amplitudes *A* and $A_v$ determined from single pulses might not be optimal for determining a warning signal amplitude. Future work will consider alternative strategies to determine perception thresholds based on circumferential stimulation with more complex warning patterns. Moreover, future studies will shift the focus from absolute threshold values to detection outcomes, i.e., whether a warning is successfully perceived under varying conditions. In this context, it will also be important to examine how different types of work activity—such as fine versus gross motor tasks—affect the perception of electrocutaneous stimulation beyond the effect of mechanical vibration alone. Movements involving high muscle activity (e.g., drilling, sanding, polishing), varying body posture, friction from work clothing, and cognitive distraction during typical occupational tasks may significantly alter signal detectability and are therefore subject to future investigation.

Temperature showed a significant influence on perception thresholds (for conditions DC, DW, and WC). A plausible explanation could be the activation of the thermoreceptors. Especially in cold environments, the additional activation of mechanoreceptors (especially Meissner’s corpuscles) due to shivering, a body’s own reaction to the cooling of the body, could have influenced perception. The higher perception thresholds in cold environments could be associated with a decreased nerve conduction velocity, which decreases by about 5 % per degree Celsius^22–24^. As the temperature increases, however, the stimulus is conducted faster. The nerve conduction velocity increases by about 2.4 m/s per ºC^25,26^, which could explain the lower perception thresholds in warm environments. Attention and intolerance thresholds were not influenced by temperature changes. Also, no threshold differences were found in dependence of the humidity. However, in humid environments, the intolerance thresholds were often above the stimulation limit of 25 mA. The effect was most pronounced in the wet-warm environment. Here, the intolerance thresholds of 23 participants were above the stimulation limit compared to 16 participants in the dry-cold environment. An explanation for this could be the increased skin temperature and moisture, which led to increased sweating on the skin in the wet-warm environment. This could have also led to a decreased transition impedance between the skin and the electrode.

Possibly the exposure times chosen for this study were too short. It cannot be ruled out that threshold shifts occur with longer exposure. In our study, we observed a tendency for slightly increasing threshold values over time inside the climate chamber. This effect was equally observable under all four climate conditions. The values of measurement cycle 1 were compared to measurement cycle 3 and averaged across all 4 electrode positions and the four climate conditions DC, DW, WC, and WW. On average, the perception threshold increased in 66% of all participants, the attention threshold in 69%, and the intolerance threshold in 35% of participants. For a few participants, the some threshold values increased by up to 40%. The chosen investigation duration of 47 min in the respective climatic conditions might need to be increased in future studies. Whether there is also a connection between increasing values for skin moisture and skin surface temperature must be examined more closely in further studies with longer exposure times.

No systematic differences were found regarding reference threshold determinations outside the climate chamber. When comparing the reference measurements from day 1 and day 2, it was noticed that the intolerance threshold was reached less frequently on the second day. It cannot be ruled out that this is related to a possible habituation effect. In order to compare the thresholds obtained from the study group at TU Ilmenau (electrode position 3) and the study group at TU Dresden (electrode position B), unpaired Wilcoxon tests were performed for perception, attention, and intolerance thresholds. The first reference threshold determination of day 1 at TU Dresden was chosen for comparison with the reference thresholds obtained at TU Ilmenau. The muscle twitch threshold was not recorded at TU Ilmenau. The test revealed statistically significant differences for the participants between TU Dresden and TU Ilmenau only for the perception threshold ($p \ll 0.05$), where the perception threshold was smaller in the study group of TU Dresden. A possible reason for that might be the difference in the reference threshold determination procedure. At TU Ilmenau, the threshold determination at electrode pair 3 was repeated 10 times. In contrast, the threshold determination at electrode pair B was repeated 3 times at TU Dresden as the thresholds were determined also at positions A, C, and D. Habituation effects were not investigated in study 1 as each threshold was determined only once, except for the reference threshold determination, but there the mean values of the last three thresholds were used for further processing. The comparison with our previous study^7^ showed that median perception, attention, and intolerance thresholds at electrode pair 3 of 3.5 mA, 6.9 mA, and 13.85 mA were smaller compared to the values of study 1 with 4.1 mA, 9.13 mA, and 18.16 mA. In our previous study, the reference threshold determination was repeated minimum 3 times until a stabilization of the thresholds was observable by the operator. In contrast, in study 1, the threshold determination at electrode pair 3 was repeated 10 times for all participants, which might partly explain the differences between the two studies. Further, it should be noted that in studies 1 and 2 threshold determinations were not stopped if muscle twitches occurred, which was the case in our previous study^7^. This might also explain the differences between attention and intolerance thresholds between study 1 and the previous study. For study 2, the median perception threshold of 3.05 mA was smaller than in our previous study. The attention and intolerance thresholds were larger with 9.4 mA and 17.4 mA. In the previous study^7^ we determined the thresholds at electrode pair 3 multiple times during the course of the experiment which took place at the same day and found no differences between the thresholds.

The median muscle twitch threshold was larger at higher temperatures compared to lower temperatures for electrode pair B and for electrode pair D during the dry climate condition. The small difference could be partly explained by the cooling-mediated increase in the calcium sensitivity of myofibrillar activation^27^. Muscle twitching occurred more frequently under all four climatic conditions than at room temperature, but generally did not pose a problem as it was only weakly pronounced and was not classified as disturbing by the participants. During the presentation of the warning pattern, unavoidable muscle twitches occurred (cf. Fig. 5). It cannot be distinguished if the increasing values of alertness, discomfort, and urgency originated from the perception of the electrical stimulation signal and/or the accompanying muscle twitches. While slight muscle twitches are tolerable for occupational warning systems, stronger twitches might interfere with the working tasks. Thus, an evaluation of the muscle twitch strength during working tasks is required for future studies. Additionally, electrode shape optimization strategies could be considered to reduce muscle twitches.

The observed changes in perception and muscle twitch thresholds for the climatic conditions were small. Additionally, there were no significant changes observed for attention and intolerance thresholds. This is advantageous for a future personal warning system, as the approach appears to be robust against external environmental influences, especially in the expected operating range between the attention and intolerance threshold. In contrast, the increasing thresholds observed with increasing vibration amplitude and frequency need to be considered in the ongoing design of the electrocutaneous warning system e.g. by adjustable stimulation parameters. However, since fixed threshold amplitudes may not be ideal for practical implementation, future systems will focus on detection outcomes—whether a warning is perceived or not—under varying conditions.

Both studies showed sex-specific differences with smaller perception and attention thresholds for women compared to men. The sex differences at the attention threshold were not significant for all Vibroshaper conditions, repeated threshold determinations, and climate chamber conditions, which can be explained by the relatively high inter-individual variability for these thresholds. Women reached the intolerance threshold ($\le$25 mA) more often than men (cf. Table S1-3). These results were comparable to other studies in the field of electrocutaneous stimulation^28,29^. The sex differences might be partially explained by sex-related physiological factors such as differences in subcutaneous fat tissue^30^, with women generally having a higher proportion of subcutaneous fat than men. Differences in the thickness of the dermis may also contribute, as men typically have thicker dermal layers than women^31^. Sweat gland characteristics differ as well: women tend to have a higher sweat gland density^32^, but men usually exhibit higher sweat gland activity^33^. Morphometric studies showed that women generally have a higher density of epidermal nerve fibers compared to men^34^, which may explain the higher perception thresholds observed in male participants. In addition, hormonal influences such as estrogen levels may modulate sensory sensitivity^35^. Psychosocial factors, such as attentional focus on the stimulus or differing expectations, may also contribute to greater response readiness in women. Additionally, female participants showed less muscle twitching than male ones (cf. Fig. 5). This difference might be explained by the fact that men show larger muscle cross-sectional areas for the biceps brachii than women^36^. Additionally, there are sex-specific differences in muscle kinetics and fiber-type composition^37^. These sex-specific differences and the high inter-individual variability originating from other factors require individually adjustable operating ranges for the future warning system.

The two studies realized at TU Ilmenau and at TU Dresden had certain limitations. We only explored vertical pairs of electrodes and used a fixed electrode size due to practical constraints. Additionally, the age distribution of our study groups was dominated by young participants. Subsequent studies will aim to include a more balanced age distribution between 18 and 65 years old, considering previous findings that suggest age-related effects on perception thresholds in electrocutaneous stimulation studies^29,38^. The use of TENS electrodes in both studies was primarily motivated by their low and consistent impedance, which ensured stable signal transmission under controlled experimental conditions. However, their material properties present limitations for long-term or real-world applications. Specifically, the hydrogel layer used for adhesion can dry out over time, particularly in environments with elevated temperature or low ambient humidity, leading to a decrease in skin contact quality and increased electrode impedance. Conversely, in high-humidity or high-sweat conditions—such as during physical activity or in warm climates—excess moisture can lead to gel saturation, slippage, or detachment, which in turn compromises the reliability of warning signal delivery. Furthermore, environmental temperature fluctuations can affect both the viscosity of the gel and the skin-electrode interface, adding another layer of variability. These factors collectively reduce the reliability and longevity of the system in wearable, day-to-day use. For future implementations, especially in wearable and long-term application scenarios, alternative electrode technologies such as dry or textile-based electrodes will be explored. These can offer improved breathability, mechanical stability, and long-term comfort, and might be better suited to dynamic environmental conditions and repeated use.

## Conclusions

We conclude that future warning systems should allow for individually adjustable operating ranges of the warning amplitudes because of the high inter-individual variability in perception, attention, muscle twitch, and intolerance thresholds both under rest and vibration conditions. Based on the high inter-individual reactions to the warning pattern presentation, we conclude that an adjusted threshold determination procedure using the actual warning signal instead of single pulses should be used in the future. The influence of muscle twitches during work tasks needs to be investigated in future studies to find out whether optimization strategies are necessary to reduce muscle twitches. In addition, changes in electrocutaneous perception during different working postures and body movements need to be examined in more detail. For practical use, it must also be examined whether (work safety) clothing worn over the personal warning system leads to impairments in warning signal perception. Perception thresholds slightly increased with decreasing temperature. Muscle twitch thresholds slightly increased with increasing temperature. We conclude that the climatic conditions within the evaluated range are of minor influence on the operating range when exposure times are less than one hour. Longer exposure times could lead to threshold shifts and thus need further experiments.

## Supplementary Information

Supplementary Information.

## Data Availability

For any queries or data requests related to this study, please contact the corresponding author E.M.D (eva-maria.doelker@tu-ilmenau.de). The experimental data supporting the publication results are available on https://doi.org/10.5281/zenodo.15974997.
