# Supplementary material for: Vibration, temperature, and humidity influence the perception of electrocutaneous stimulation for occupational warning
Source: Sci Rep. 2025 Nov 4;15:38471. doi: 10.1038/s41598-025-25166-2 (PMC12586676; doi:10.1038/s41598-025-25166-2)
Supplement: Supplementary file 1 — Supplementary Information. [file 41598_2025_25166_MOESM1_ESM.pdf]

Supplement

Threshold / Vibration amplitude and Vibration frequency	Perception		Attention		Intolerance	
Sex	f	m	f	m	f	m
Rest	100 %	100 %	100 %	100 %	70 %	53 %
Vibration amplitude 2 mm & Vibration frequency 8 Hz	100 %	100 %	100 %	98 %	63 %	43 %
Vibration amplitude 5 mm & Vibration frequency 8 Hz	100 %	100 %	98 %	84 %	60 %	33 %
Vibration amplitude 8 mm & Vibration frequency 8 Hz	100 %	94 %	98 %	76 %	40 %	25 %
Vibration amplitude 2 mm & Vibration frequency 9.5 Hz	100 %	98 %	98 %	94 %	58 %	37 %
Vibration amplitude 5 mm & Vibration frequency 9.5 Hz	100 %	94 %	98 %	80 %	51 %	24 %
Vibration amplitude 8 mm & Vibration frequency 9.5 Hz	100 %	88 %	93 %	71 %	37 %	18 %

Table S1. Percentage of the $n = 43$ female and $n = 51$ male participants that reached (≤25 mA) the thresholds during rest and under vibration in dependence of the vibration amplitude and vibration frequency.

		Day 1						Day 2					
Electrode pair	Threshold determination no.	1		2		3		1		2		3	
	Sex	f	m	f	m	f	m	f	m	f	m	f	m
A	Muscle twitches	55 %	75 %	60 %	81 %	50 %	81 %	50 %	84 %	50 %	87 %	55 %	91 %
B		50 %	66 %	55 %	66 %	60 %	78 %	55 %	56 %	50 %	59 %	60 %	62 %
C		50 %	75 %	45 %	78 %	60 %	72 %	45 %	91 %	50 %	87 %	55 %	87 %
D		45 %	59 %	55 %	59 %	60 %	62 %	35 %	56 %	50 %	69 %	55 %	81 %
A	Intolerance threshold $A_i \leq 25$ mA	80 %	62 %	80 %	56 %	85 %	59 %	75 %	59 %	75 %	62 %	75 %	50 %
B		80 %	44 %	70 %	47 %	80 %	44 %	70 %	50 %	70 %	47 %	65 %	47 %
C		75 %	59 %	80 %	62 %	80 %	56 %	60 %	47 %	65 %	44 %	65 %	44 %
D		75 %	53 %	80 %	53 %	85 %	53 %	75 %	50 %	70 %	44 %	70 %	41 %

Table S2. Percentage of the $n = 20$ female and $n = 32$ male participants experiencing muscle twitches and reaching (≤25 mA) the intolerance threshold for the reference threshold experiments.

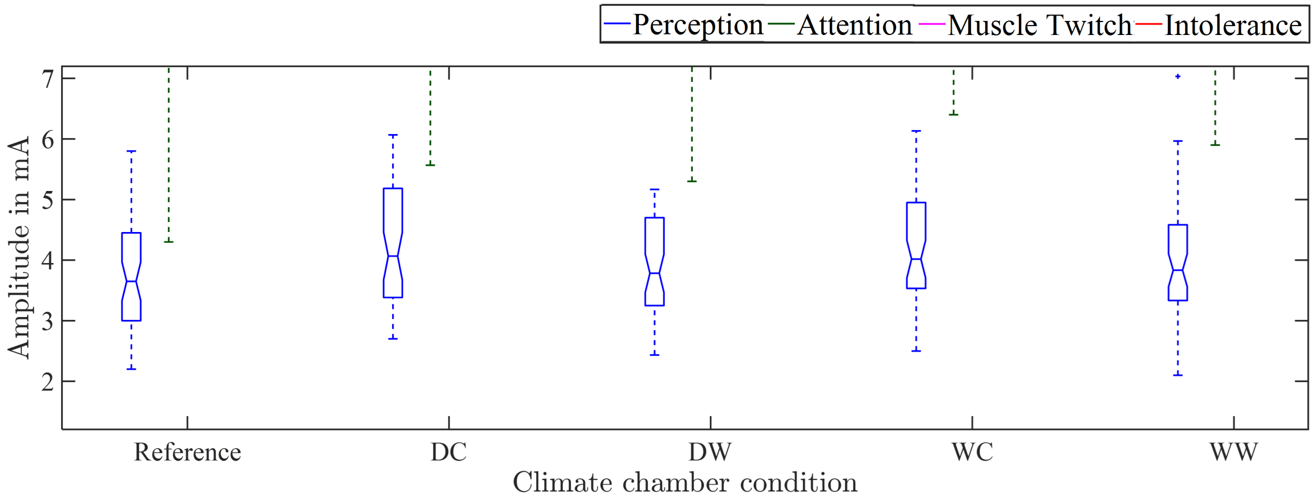

Figure S1. Thresholds at electrode pair B for climatic conditions outside of the climate chamber (Reference), dry-cold (DC), dry-warm (DW), wet-cold (WC), and wet-warm (WW) enlarged from Fig. 7 to view perception threshold.

Electrode pair		Reference		Dry cold		Dry warm		Wet cold		Wet warm	
	Sex	f	m	f	m	f	m	f	m	f	m
A	Muscle twitches	55 %	75 %	50 %	89 %	47 %	84 %	55 %	83 %	60 %	81 %
B		50 %	66 %	53 %	60 %	55 %	60 %	60 %	66 %	53 %	57 %
C		50 %	75 %	38 %	79 %	45 %	78 %	52 %	74 %	55 %	69 %
D		45 %	59 %	45 %	72 %	37 %	68 %	50 %	60 %	43 %	58 %
A	Intolerance threshold $A_i \leq 25$ mA	80 %	62 %	80 %	55 %	80 %	44 %	75 %	49 %	72 %	40 %
B		80 %	44 %	73 %	48 %	68 %	42 %	68 %	36 %	67 %	35 %
C		75 %	59 %	70 %	44 %	55 %	39 %	73 %	32 %	67 %	40 %
D		75 %	53 %	65 %	48 %	65 %	51 %	73 %	44 %	67 %	42 %

Table S3. Percentage of the $n = 20$ female and $n = 32$ male participants experiencing muscle twitches and reaching ($\leq$25 mA) the intolerance threshold for the first reference threshold experiment and inside the climate chamber.

Environmental condition	skin surface temperature	skin surface moisture
RTE (d1/r1)	31.6 ± 3.1 °C	51.3 ± 10.9% RH
DC	25.7 ± 3.5 °C	39.3 ± 13.1% RH
WC	25.2 ± 2.9 °C	58.1 ± 11.0% RH
DW	37.0 ± 2.0 °C	64.1 ± 19.7% RH
WW	36.9 ± 1.7 °C	82.9 ± 11.7% RH

Table S4. Skin surface conditions for $n = 52$ participants (f=20/m=32) during the first reference threshold experiment (RTE day 1, repetition 1) and in the climate chamber under dry-cold (DC), dry-warm (DW), wet-cold (WC) and wet-warm (WW) conditions. Skin surface temperature and skin surface moisture are given as mean ± standard deviation.
